# Supplementary material for: Are carbon emissions trading and green financial instruments synergistic? -Comprehensive quantitative research based on content analysis
Source: PLoS One. 2024 Mar 7;19(3):e0298601. doi: 10.1371/journal.pone.0298601 (PMC10919601; doi:10.1371/journal.pone.0298601)
Supplement: S1 Data — (ZIP) [file pone.0298601.s001.zip › Data/地级市Z_F工作报告.zip!/╕╩╦α/╝╬╙°╣╪╩╨/└┤╫╘╣1⁄2╓┌║┼í╛┬φ┐╦╩2╛▌═°í┐_2017.docx]

各位代表：

　　现在，我代表市人民政府，向大会作工作报告，请予审议，并请各位政协委员和列席人员提出意见。

　　一、2016年工作回顾

　　过去一年，面对严峻复杂的经济形势和艰巨繁重的发展任务，在省委、省政府和市委的坚强领导下，全市上下认真贯彻落实党的十八大和十八届三中、四中、五中、六中全会精神，深入学习贯彻习近平总书记系列重要讲话精神，牢固树立和贯彻落实新发展理念，积极应对经济下行压力，突出经济转型、突出改革创新、突出补齐短板，优化发展思路，坚定发展信心，扎实推进稳增长、促改革、调结构、惠民生、防风险各项工作，较好地完成市九届人大五次会议确定的主要目标任务，实现了“十三五”良好开局。

　　——经济运行缓中趋稳。主要经济指标均保持在合理区间，全市实现生产总值153.4亿元，经济增速由第一季度的1.2%回升到第四季度的8%，按可比价计算全年增长7.3%，一二三产业分别增长5.6%、3.1%、13.4%。通过坚持不懈做精一产、做强二产、做大三产，三次产业结构由上一年度的2.2∶57∶40.8调整为2.9∶39.3∶57.8，第三产业实现历史性突破。固定资产投资160.4亿元，增长11.3%。社会消费品零售总额60亿元，增长9.2%。居民消费价格涨幅1.2%，控制在3%以内。一般公共预算收入完成17.09亿元，增速由上一年度的-12.9%恢复性增长到12.1%。城乡居民人均可支配收入稳步增长，分别达到33540元、16462元，增长9.2%、7.1%。全市经济发展总体呈现缓中趋稳、稳中有进的态势。

　　——工业转型步伐加快。坚持从供给侧入手，着力改造提升传统优势产业，积极培育新兴产业，工业经济止跌企稳。实现规模以上工业增加值48.74亿元，增速由年初的-9.7%回升到年末的3.1%，全年增长2.6%。传统产业缓中趋稳，规模以上工业企业实现利润总额35.7亿元，同比增盈124.8亿元。酒钢集团扭亏为盈，实现盈利20.3亿元，并安全兑付128亿元到期债券，化解了金融风险。发展后劲稳步增强，围绕钢、铝两大千亿级产业链，全力推进延链、补链、强链项目建设，宏汇150万吨煤炭分质利用、酒钢钢结构制造、嘉策铁路扩能改造、330KV宏丰开关站改造、索通预焙阳极二期及余热综合利用等重点项目基本建成，天成彩铝40万吨绿色短流程铸轧铝深加工、粉矿悬浮磁化焙烧选矿改造工程等重点项目顺利推进。循环经济取得新进展，全年工业固废综合利用率达到61.8%，单位GDP能耗和万元工业增加值用水量均下降6.7%，万元工业增加值能耗下降7.47%，工业园区成功创建国家级低碳示范园区和循环化改造示范试点园区。工业经济动能转换势头良好，结构优化成效明显。

　　——文化旅游融合发展。全年接待旅游人数702万人次，全社会旅游收入45.3亿元，分别增长23.2%和25.9%；文化产业增加值增长15.1%。着力推进文物系统管办分离、事企分开改革，大景区管理体制改革方案获得批准，丝路（长城）文化研究院和大景区管理委员会顺利运行。谋划推进丝绸之路文化博览园、世界文化遗产公园（花卉博览园）、峪泉古镇古街、长城保护连接道路、文化旅游产业创意园5大项目建设，丝博园累计完成投资11.7亿元，长城保护连接道路工程建设接近尾声；长城本体保护工程全部竣工，玉龙湾文化生态园、游客集散服务中心、智慧旅游建设、房车露营地、航空博物馆、文化遗产历史“再现工程”等重点项目稳步推进，《丝绸之路彩绘艺术大观》和《嘉峪关文化丛书》两大项目启动，戈壁大峡谷获批省级地质公园。扎实开展全域旅游基础性工作，全域旅游发展规划、关城大景区建设性详规、新城魏晋墓群文物保护规划和可移动文物普查等工作进展顺利，成功入选第二批国家全域旅游示范区试点单位。商贸物流业快速发展，天诚广场购物中心建成投运，雄关大厦、天城美居等重点项目顺利推进，铁路物流园区项目获批，嘉峪关国际航空口岸项目列入国家“十三五”发展规划。

　　——城乡发展统筹推进。强化城市规划管理，《嘉峪关市城市总体规划（2016—2030）》评估与修编工作全面完成，编制了峪泉镇“多规合一”试点规划、棚户区改造修建性详规、城市地下综合管廊等专项规划。协调推进基础设施建设，南市区道路、集中供热、胜利路提升改造、双拥路口公铁立交、汽车南站、综合客运枢纽等项目进展顺利，G312线嘉峪关至清泉公路、S305嘉峪关至祁丰公路，断山口至树窝井、部队训练场至黑鹰山战备公路等重点交通建设项目有序推进。全年财政投入“三农”资金1.95亿元，持续改善农民生产生活条件。新建和改造农村道路146公里，标准化日光温室105座、新建和改扩建养殖小区9个，实施高效农田节水技术推广7万亩、土地开发整理1000亩。积极开展国家建制镇示范试点和“美丽乡村”建设，农村教育、医疗、低保、养老、公共文化、环境卫生等领域一体化建设不断加强。酿酒原料、高效制种、苗木花卉、戈壁枸杞等特色产业不断壮大，农业观光、生态采摘、休闲服务等新产业、新业态不断发展，累计建成乡村旅馆、民俗客栈37家，星级农家乐73家。以文殊镇恒翔现代农业示范中心、新城镇野麻湾农业发展示范园区为代表的“企业+基地+合作社+农户”产业化运营模式初具规模。农村土地产权制度改革有序推进，土地承包经营权确权登记与合同签订率达到97.6%，土地流转率43.7%。深入开展“联村联户”行动，投入帮扶资金2100万元，组织干部进村入户1.1万人次，帮办实事720件。

　　——创新动能明显增强。积极实施创新驱动发展战略，制定出台深化人才体制机制改革若干意见、“十三五”科技创新规划、科技创新发展若干措施、科学技术奖励办法、大众创业万众创新实施方案。优化科技资源配置，财政投入科技经费1460万元，带动企业研发投入4.6亿元，组织实施科技计划项目62项，研发高强度低合金钢、优质N234炭黑等新产品23个。全市新增省级以上科技创新平台5个，大友企业公司被认定为国家知识产权优势企业。酒钢集团组织开展科技项目222项，取得国家专利授权131项，新产品研发33项，其中锌铝镁合金产品及其生产技术填补国内空白，汽车结构用热轧高强钢、冷轧高强钢、汽车尾气管用钢打入汽车制造企业，2205双相不锈钢成功进入国内换热器制造领军企业。全市研发经费支出占GDP的比重为4.32 %，科技进步对经济增长的贡献率提高到48.6%，综合科技进步水平指数在全省排名第4位；每万人口发明专利拥有量提高到4.39件，居全省第3位。着力推进大众创业、万众创新，向城乡各类人员和小微企业提供小额贷款担保3342万元，全市发展众创空间7家，认定国家级中小企业公共服务示范平台1家，省级众创空间及孵化器3家，为中小企业孵化中心成功申报省级补助资金450万元。

　　——改革活力持续释放。认真落实“三去一降一补”任务，酒钢集团压减榆钢100万吨生铁和140万吨粗钢产能，本部铁、钢、材产量均减少10%以上；全市铁合金减少28.8%。结合棚户区改造加大货币化安置力度，商品住宅去化周期由年初的19个月下降至年末的17个月。阶段性降低企业职工社会保险费率，为企业减负3510万元，落实34户企业稳岗补贴2550余万元，为22户困难企业减免土地使用税和房产税1500余万元。配合酒钢集团争取国家建设基金3.17亿元，落实化解钢铁过剩产能职工分流安置中央奖补资金2.26亿元。扎实推进“放管服”改革，累计取消行政审批事项69项，清理规范行政审批中介服务事项6项；投资项目报建审批事项由45项减少到27项。完善“三张清单一张网”建设，41个政府部门和直属事业单位的10类行政权力事项初步实现网上行权。推进行政审批和公共资源交易“全流程”监管，电子招投标公共服务、交易和行政监督平台投入使用。推进“双随机一公开”监管体制改革，相关单位“一单两库”初步建立。推进商事制度改革，简化注册登记手续，市场准入由“先证后照”改为“先照后证”，启动市场主体简易注销登记试点，加快实现注册登记全程电子化，企业登记注册时间平均缩短60%以上，企业创业准入的制度性成本不断降低。全面推开建筑、房地产、金融、生活服务业“营改增”试点工作，全年减轻纳税人税负3970余万元，减税面达到98%。不动产统一登记工作实现“停旧发新”目标，登记数据完成国家汇交。深化投融资体制改革，鼓励支持金融机构创新开发“银政通”、“转贷基金”、“助保贷”等业务，稳步推进中小企业信用担保、下岗失业人员小额贷款担保和农业信用担保机构整合工作。截止2016年末，全市各项贷款余额477.69亿元，比年初增长12.3 %；西部重工在“新三板”正式挂牌，6家企业纳入全省重点后备企业培育资源库，67户企业在省股交中心挂牌，实现融资9.45亿元。推动非公经济健康快速发展，制定出台“25条”扶持和促进非公有制经济加快发展试行办法，实施小微企业互助贷款工程，落实风险补偿金2000万元，累计发放贷款6550万元；建立健全“三联秘书”服务机制，发展“个转企”164户。

　　——开放合作成果丰硕。成功举办国际铁人三项戈壁挑战赛、第五届国际短片电影展、首届丝绸之路国际房车博览会和丝绸之路长城戈壁马拉松赛等各类节会赛事，城市知名度和影响力不断提升。积极组织参加敦煌国际文博会、兰洽会、广交会等节会，进一步加大招商引资力度，全年招商到位资金184亿元，同比增长12.2%。先后与中铁二十二局、甘肃民航机场集团、甘肃建投、上海飞和集团、上海交大教育集团等签订战略合作协议，着力推进多功能专用汽车研发制造基地、公共飞行基地、城市基础设施、公共社会事业等项目建设。扎实推进10万辆多功能整车制造项目，首台试装车辆成功下线。酒钢集团成功签订收购俄铝牙买加氧化铝厂股权协议，宏兴股份与印尼东昇公司签订合作框架协议；“嘉峪关”号国际货运班列常态化运行，澜驰汽配等企业产品成功打入中亚、中欧国家。全年完成外贸进出口总额17.65亿元，增长50.7%。

　　——绿色发展成效明显。贯彻绿色发展理念，实施“生态立市”战略，着力推进“蓝天、碧水、净土”工程。城区绿地率和绿化覆盖率达到38.1%和39.2%，人均公园绿地面积达到35.9平方米，成功创建国家园林城市，被列为省级水生态文明建设试点城市。切实加大环境保护力度，全面实施大气、水、土壤污染防治“三大行动”，工业污染治理、燃煤锅炉综合整治、扬尘污染防治、煤场综合整治、黄标车及老旧车辆淘汰等工作成效明显；圆满完成省政府下达的20项污染减排任务，污染减排各项指标基本完成，空气质量优良天数达到315天，优良率86.1%；中央第七环境保护督察组转办27件信访投诉案件全部办结。大力推行清洁生产和循环经济，全面完成国家循环化改造示范试点任务。扎实开展环境卫生百日综合整治，“定位划线”、“五小六乱”治理、交通秩序整治等“八大”专项整治行动取得阶段性成效。把创建全国文明城市作为惠民、利民的系统工程持续推进，市民文明素质和社会文明程度不断提升。

　　——民生保障持续提升。全年民生及社会事业支出17.82亿元，占一般公共预算支出的73.4%。实施积极就业政策，组织各类就业创业技能培训4248人次，开展职业技能鉴定3263人，组织劳务输转7198人。安置就业困难人员、零就业家庭人员248人，发放灵活就业社会保险补贴470余万元；新增城镇就业8025人，高校毕业生就业率85%，城镇登记失业率控制在2.75%。持续提高社会保障水平，机关事业单位人员全部纳入养老保险覆盖范围，农民工工伤保险覆盖率不断提高。整合城乡医疗保险，基本实现省内异地安置退休人员就医直接结算；出台医疗救助实施办法，重特大医疗救助病种从26种扩大到50种。城乡低保、农村五保供养及城市三无人员保障标准不断提高。积极保障农民工权益，受理农民工投诉举报案件516件，结案率96%，涉案金额2879万元。推进医疗与养老机构融合发展，完善居家养老服务平台，在册老人近1.8万人；残疾人康复托养服务中心建成投运，实现残疾人康复、托养、健身、教育“四位一体”服务新模式。坚持教育优先发展，实施教育基本建设项目17项，完成投资8060万元，启动和续建校舍建筑面积9.8万平方米；投资2500多万元推进“全面改薄”，教育均等化水平不断提高；学前三年毛入园率、义务教育巩固率、高中阶段毛入学率分别达到97.8%、99.8%和95.1%，均位居全省前列。着力补齐医疗卫生短板，累计投入3.57亿元，实施市第一人民医院门急诊楼、儿童专科病区、中医院综合楼等项目建设，酒钢医院危旧病房改造项目竣工投运；认真落实药品采购配送制度，分级诊疗和多点执业改革有序推进。严格落实“党政同责、一岗双责、失职追责”安全生产责任体系，深刻汲取酒钢西沟矿“8·16”重大火灾事故教训，深入开展“百日安全生产大检查”活动，累计整改安全隐患3111处，整改率85.1%。推动社会治理方式创新，以全省立体化社会治安防控体系建设试点为契机，不断完善社会治理体系，推进综治信息平台联网应用；健全民生诉求联动处置机制，“12345”民生热线全年受理公众诉求2.98万件，同比增长43.9%；加大金融诈骗、电信诈骗、非法集资等各类违法犯罪行为打击力度，社会大局保持和谐稳定。七项惠民实事基本完成，实施棚户区改造4394套、新增保障家庭住房438户，完成计划任务的128%、216%；提高城乡居民低保标准10%、为残疾儿童提供康复治疗服务、实施残疾人家庭无障碍改造、新建改造8所公共卫生间全面完成；建设2所幼儿园完成招投标，新建5个城市标准化社区服务中心积极推进。

　　——自身建设不断加强。过去一年，我们牢固树立“四个意识”，扎实开展“两学一做”学习教育，贯彻落实两《准则》、三《条例》，着力建设法治政府、效能政府、廉洁政府。全面推行政务公开，推进行政决策科学化、民主化、法治化，不断强化政府公信力。自觉接受人大及其常委会的法律监督、政协民主监督和社会各界监督，107件人大代表建议、103件政协提案全部按期办结。加强审计监督，完成审计及其调查项目39项，查处违纪违规和管理不规范资金8992余万元，上缴财政资金70余万元，核减工程造价5410万元；对全省审计跟踪问责事项涉及到的28个单位124名责任人进行了问责。深入推进作风建设，突出重点领域、重点任务、重点事项，加大督查力度，强化跟踪问效，全面推动工作落实。从严控制各类会议活动，压缩会议规模和时间；严控文件、简报数量，政府系统全面推行无纸化公文传递，办公效率明显提高。严控一般性支出，全市“三公”经费开支较上一年度下降2.7%。党政机关公车改革全面完成。认真履行“一岗双责”主体责任，切实加强党风廉政建设，不断强化行政监察，共受理信访举报160件，立案21件，结案19件，给予党纪政纪处分22人，涉嫌犯罪移送司法机关处理9人。

　　各位代表！过去一年，面对经济下行压力持续加大的严峻形势，取得这样的成绩实属不易。这些成绩的取得，是省委、省政府亲切关怀和市委正确领导的结果，是市人大、市政协、嘉峪关军分区、酒钢集团公司、中核四〇四有限公司及驻嘉各单位大力支持的结果，是全市各族人民团结拼搏的结果。在此，我代表市人民政府，向全市各族人民、人大代表、政协委员、各民主党派、工商联、无党派人士和人民团体，向驻嘉部队、武警官兵和驻嘉各单位，向所有关心支持嘉峪关发展的社会各界朋友，表示衷心的感谢，并致以崇高的敬意！

　　各位代表，在肯定成绩的同时，我们也清醒地看到，全市经济社会发展中还存在一些不容忽视的困难和问题。主要表现在：经济结构比较单一，抵御市场风险能力依然较弱；工业投资增长乏力，民间投资后劲不足；经济企稳基础不牢，下行压力仍然较大；创新发展动力不足，人才资源短缺；发展环境不够宽松，“放管服”改革仍需深化；补齐全面建成小康社会短板的时间紧、任务重；城市精细化管理任务艰巨，建设管理机制亟需理顺；一些干部能力素质不过硬，责任担当意识不强。这些问题，我们要努力用改革的思路和创新的办法，切实加以解决。

　　二、2017年主要任务

　　今年是贯彻落实市第十一次党代会和市人大十届一次会议精神的第一年，也是供给侧结构性改革的深化之年。中央将召开党的十九大，我省也要召开第十三次党代会，做好今年政府各项工作意义特殊而重大。我们必须全面贯彻党的十八大和十八届三中、四中、五中、六中全会精神，认真落实中央、省委和市委经济工作会议精神，以邓小平理论、“三个代表”重要思想、科学发展观为指导，深入学习贯彻习近平总书记系列重要讲话精神特别是视察甘肃时“八个着力”的指示要求，统筹推进“五位一体”总体布局，协调推进“四个全面”战略布局，坚持稳中求进工作总基调，坚持以新发展理念为引领，以提高发展质量和效益为中心，以推进供给侧结构性改革为主线，紧盯实现“两个率先、三个翻番、四个提升”奋斗目标，着力深化改革创新、优化经济结构、扩大对外开放、保障人民生活，全面做好稳增长、促改革、调结构、惠民生、防风险各项工作，促进经济平稳健康发展和社会和谐稳定，以优异的成绩迎接党的十九大和省第十三次党代会胜利召开。

　　今年经济社会发展的主要预期目标是：生产总值增长8%左右，固定资产投资增长12%左右，社会消费品零售总额增长9.5%，一般公共预算收入增长6%，城乡居民人均可支配收入增长9%，居民消费价格指数涨幅控制在3%以内，单位生产总值能耗和主要污染物排放完成省级下达的控制指标。

　　为实现上述目标，我们重点抓好十个方面的工作。

　　（一）突出创新驱动，全面培育发展动能。牢固树立人才资源是第一资源、科学技术是第一生产力、大众创业万众创新是培育经济发展动力之源、新一代信息技术是推动发展重要引擎的意识，大力实施创新驱动发展战略，加速产业转型、动能转换。

　　优先实施人才发展战略。认真落实深化人才发展体制机制改革若干意见“60条”，坚持问题导向，正视突出短板，努力破解制约人才优先发展的体制障碍，创新育才、引才、用才、留才机制，建设人才强市，集聚更多的领军人才、高技能人才和高水平创新创业团队，为各类人才干事创业、成就梦想创造更多的机会和环境，为促进经济社会转型发展提供人才保障和智力支撑。

　　加大科技创新支持力度。全面落实“十三五”科技创新规划“2469”重点任务、加快科技创新发展若干措施“35条”和新修订的科技奖励办法“22条”，实施科技创新“苗圃”计划，设立科技创新创业“资金池”，实行科技创新券制度，支持中小微企业和创新创业团队发展壮大，充分发挥企业创新的主体作用；建立区域性科技成果交易服务平台，争创省级、国家级可持续发展试验区和国家知识产权试点城市；力争研发经费支出占GDP的比重达到4.4%，科技进步贡献率达到50%以上。

　　深入推进大众创业万众创新。全面落实大众创业万众创新实施方案“45条”，通过打造创新创业广场，提供“双创”资金保障，扶持众创空间发展，开展技能创业培训，支持大学生创新创业，鼓励企事业单位工作人员离岗创业，完善财税支持政策，建立协同推进工作机制等一系列行之有效的措施，着力打造发展新引擎、增强发展新动力。坚持办好“科技双创活动周”、科技创新大赛、就业指导及招聘活动，持续营造创业创新的良好环境。

　　强力推动信息化建设。坚持问题导向、需求驱动、注重实效原则，按照成立一个强有力的协调领导机构，建立一个统一、协调、有序、高效的执行机构，搭建一个高起点、高标准、高效率的综合数据交换与共享平台，组建一支专业化的队伍，成立一个市场化运营的服务平台“五个一”结构体系，推进信息资源整合，加快新一代信息技术应用和智慧城市建设，着力发挥信息化对经济社会发展的驱动引领作用。

　　（二）加快转型步伐，推动工业提质增效。以推进供给侧结构性改革为主线，加快产业集群发展、高端发展、绿色发展，推动传统产业新型化、新兴产业规模化、支柱产业多元化。

　　紧紧围绕钢、铝两条千亿级产业链，持续推进延链、补链、强链项目建设，发展壮大战略性新兴产业。全力支持酒钢集团转型发展，服务好新型金属复合材料产业化、高炉优化升级改造、煤炭分质利用、绿色短流程铸轧铝深加工、牙买加铝产业基地等重点项目建设，加快提升钢铁、电解铝、电力能源、煤化工四大百亿元级支柱产业发展质量。推进中威斯二期、索通公司二期、德弘矿业年产30万吨铝材加工、兴栋公司15万吨航空轻合金等一批项目建设，争取年内投产见效。认真落实支持汽车产业发展的若干意见“21条”和房车产业发展扶持办法“16条”，加快汽车装备制造业发展，努力实现“一业兴、百业旺”。力争全年战略性新兴产业比重达到5.5%。

　　把“三去一降一补”作为工业转型升级的重中之重。严格执行环保、能耗、质量、安全等法律法规和标准，认真落实省政府淘汰钢铁、水泥、电解铝、铁合金等重点行业落后产能计划，有序退出过剩产能，妥善安置人员，保持企业和社会稳定。抓紧建立园区企业准入、清退机制，“一企一策”推进停产半停产企业盘活低效无效资产，提高资产运营效率，加快清理“僵尸企业”，通过优化存量、引导增量、主动减量，切实提高投资强度和技术水平。

　　扎实开展“企业服务年”活动。强化质量发展工作，实施名牌战略；深入推进互保共建，想方设法帮助企业搭建产销对接合作平台，不断扩大工业品的市场占有率；引导、帮助企业加强科学化、精细化管理，强化内部挖潜，努力降低企业管理成本；争取资金、税收、大用户直供电、新能源就地消纳等政策支持，降低企业生产成本；进一步清理、简化、整合涉企行政事业性收费，落实各类正税清费政策，着力降低企业制度性交易成本；认真落实阶段性降低企业社保费率、稳岗补贴等一系列优惠政策，不断降低企业用工、用能、物流等经营性成本；通过调整优化信贷结构和展期、续贷等措施，帮助有潜力、有信用的企业有效降低融资成本，努力化解企业过高杠杆率；积极争取国家化解过剩产能金融债权债务问题政策，降低企业负债率，支持企业债转股。

　　（三）推进文旅融合，打造全域旅游高地。抢抓创建国家全域旅游示范区的有利时机，着力培育全域、全季、全业旅游产业，加快形成文旅商体“四轮驱动”融合发展格局，打造丝绸之路沿线重要国际旅游目的地和休闲消费型城市。

　　统筹推进重点项目建设。把关城景区5大项目建设作为推动文旅深度融合的龙头带动和示范工程，举全市之力加快建设进度，为2018年正式运营打好基础。强力推动智慧旅游、游客集散综合服务中心、自驾车旅游服务区和露营地、公共航空飞行营地、户外徒步基地、观礼古镇等项目建设；着力推进戈壁大峡谷地质公园、草湖国家湿地公园、魏晋民俗文化展览馆、黑山岩画博览园、航空博览园等重点项目前期工作，力争早日开工建设、发挥集聚效应。

　　扎实开展全域旅游试点。编制完成全域旅游发展总体规划、关城大景区建设专项规划及详规；着手编制以草湖湿地公园、魏晋墓群为代表的东北片区，以方特欢乐世界、南湖生态公园为代表的东南片区，以石关峡、黑山岩画为代表的西北片区3大景区专项规划。整合文物、文化、旅游相关职能，加快推进文化旅游管理体制改革；坚持文物保护与合理利用相结合，切实加大研究和展示利用力度；与中国社科院开展全面合作，积极举办各类主题论坛，争取人才智力支持。持续培育品牌节会赛事，办好国际短片电影展、房车博览会、铁人三项赛、国际滑翔节、长城马拉松等节会赛事，积极承接大型会议、展览、展销活动。优化整合宣传促销力量，切实加大对关城文物景区、方特欢乐世界、魏晋墓壁画、草湖湿地公园、黑山摩崖石刻、紫轩葡萄庄园、中华孔雀苑、东湖生态景区、讨赖河大峡谷、“七一”冰川10大景区景点的宣传促销力度。借助敦煌文博会的集聚带动效应，加大与酒泉、张掖、东风航天城、额济纳旗、海西州、哈密等周边地区合作力度，以拓展“七一”冰川探险游、文殊山石窟游、镜铁山矿区游、核军工科技游、东风航天城游为依托，主动构建区域旅游合作体，打造全域、全民、全业、全景和全时的“五全融合”旅游片区。

　　加快推动商贸物流业发展。抓住《国家西部大开发“十三五”规划》将嘉酒地区确定为次级增长区域和陆桥通道西段节点城市的有利时机，全力推动国际空港、机场改扩建、铁路物流园区、国际港务区、嘉峪关综合客运枢纽中心项目落地；加快实施嘉丰源综合大厦、一得乐商城改造，有序推进方特新天地城市综合体、南湖大厦二期、红星美凯龙家居建材市场等建设项目，提升商贸物流业发展水平。

　　（四）统筹城乡一体，补齐农村发展短板。全面贯彻落实中央一号文件精神，把推进农业供给侧结构性改革、加快培育农业农村发展新动能、推进基本公共服务均等化，作为夯实共享发展基础的着力点，全面提升农业农村发展水平。

　　持续深化农村综合改革。围绕农业增效、农民增收、农村增绿，加大农村改革力度。全面完成农村集体土地确权登记颁证工作，有序推进土地“三权分置”并行，加快土地经营权有序流转进程。推动宅基地在村集体流转和有偿退出，不断优化土地资源配置，激活农村各类要素潜力，盘活“沉睡”资产。推进农村水价改革，完善水权交易制度，健全水权交易市场。

　　加快培育特色优势产业。谋划建设农业文化旅游“三位一体”、生产生活生态同步改善、一二三产深度融合的特色村镇，着力打造“一镇一业、一村一品”产业新格局。峪泉镇主要围绕国家建制镇示范试点，抓好以旅游产业带动为龙头的新型城镇化建设；文殊镇侧重抓好戈壁生态农业产业示范园区建设；新城镇重点以规模化种养基地为基础，抓好特色优势产业培育。支持有条件的村组建设以农民合作社为主要载体、让农民充分参与和受益，集循环农业、创意农业、农事体验于一体的田园综合体；谋划开发整村推进的乡村旅游项目和创建园艺作物标准园，推进农业、林业与旅游、教育、文化、康养等产业深度融合。培育壮大农业产业化龙头企业、农民合作社、现代青年农场主等新型经营主体，聚集现代生产要素，建设“生产+加工+科技”的现代农业产业园。

　　加强农村人才队伍建设。实行教育培训、认定管理、政策扶持“三位一体”培训制度，着力培养以新型职业农民为主体的农村实用人才，紧紧围绕新型职业农民培育、农民职业技能提升，整合各渠道培训资金资源，完善部门协作、统筹安排、产业带动的培训机制。强化农业科技推广，创新公益性农技推广服务方式，引入项目管理机制，着力推行政府购买服务，支持各类社会力量广泛参与农业科技推广；鼓励建立农科教产学研一体化农业技术推广联盟，支持农技推广人员与家庭农场、农民合作社、龙头企业开展技术合作。

　　提升农村基本公共服务水平。统筹推进城乡教育一体化，通过开展对口帮扶和一体化办学、校长教师轮岗交流和培训，补齐乡村教育短板，盘活农村闲置校舍资产，避免出现“边建设、边闲置”现象。持续加强农村居民基本公共卫生服务，不断解决村卫生室和乡镇卫生院面临的紧迫问题，积极破解农村医疗卫生人才瓶颈；探索发展田园式休闲养老，将城市居家养老模式逐步向农村延伸。大力开展农村人居环境治理和美丽宜居乡村建设，全面实施“千村美丽、万村整洁”工程，开展城乡垃圾乱排乱放集中排查整治行动。深入推进“联村联户、为民富民”行动和“富而思源、富而思进”活动。

　　（五）加大改革力度，激发内生发展活力。坚持把改革贯穿经济社会发展始终，深化重点领域和关键环节改革，持续增强发展内生活力和动力。

　　深入推进“放管服”改革。完善负面清单、权力清单、责任清单制度，强化专业技术人员培训，增强“接”“管”能力，提高“放管服”的协同性和联动性；推行工程建设领域“多评合一”、“联合验收”新模式，解决程序繁、环节多、衔接不畅的问题。强化事中事后监管，推动实现“双随机一公开”监管方式全覆盖，市场监管执法和其他行政执法事项随机抽查分别达到70%和50%以上。加快构建“互联网+政务服务”体系，全面建成项目在线审批监管、信用信息交换共享、公共资源交易三大平台，推动实体政务大厅与网上服务平台深度融合，实现联审联批和“一门式”服务；通过优化服务流程，推进数据共享，推动部门间政务服务相互衔接、协同联动并向社区延伸。

　　积极推动国资国企改革。全力支持酒钢集团、中核四〇四有限公司、铁路系统等在嘉企业加快剥离办社会职能，切实做好“三供一业”分离改革工作。全面启动市属国有企业改革，对商业类国有企业发展经营无望的，通过实施破产清算、兼并重组、发展混合所有制经济等多种途径改造一批、出清一批、提升一批。对公益型国有企业，依据国有资本占有率和提供公共服务保障水平开展绩效评价，编制经营预算，实施绩效管理，加强审计监督。加快完善城投公司、文旅集团、水文化集团、经济技术开发区发展公司法人治理结构，筹备成立水务投资集团，切实提升企业治理水平和规范管理能力。

　　深化投融资体制改革。改善企业投资管理，确立企业投资主体地位，建立投资项目清单管理制度，优化管理流程，充分激发社会投资动力与活力；通过科学界定投资范围、优化投资安排方式、规范投资管理、加强投资监管、鼓励政府和社会资本合作，发挥好政府投资的引导带动作用；创新融资机制，大力发展直接融资，加快设立城市基础设施建设发展基金、文化旅游发展基金，尽快整合中小企业信用担保、下岗失业人员小额贷款担保和农业信用担保机构，完成金融服务公司整合筹建工作。

　　（六）狠抓项目建设，发挥投资支撑作用。按照筹划项目抓前期、签约项目抓落地、预备项目抓开工、续建项目抓进度、竣工项目抓投运的要求，全力抓好74项总投资近千亿元的市级领导包抓重点项目建设，充分发挥项目投资稳增长、调结构、惠民生、补短板的支撑作用。

　　加强项目谋划储备工作。紧紧围绕国家产业政策、投资导向和全省支持发展重点，加强系统研究，主动超前谋划，用好重大项目前期工作经费，强化项目论证、储备和跟进对接，积极做好三年滚动投资计划；抓好专项建设基金项目谋划储备和申报工作。在主动融入“一带一路”建设、区域性综合交通枢纽、传统产业转型升级、新兴产业培育发展等方面，谋划一批起点高、体量大、带动性强、市场前景好的项目。围绕延长产业链条，积极承接产业转移，大力开展全产业链招商、节会招商、精准招商。加强部门联动，靠实招商责任，强化跟踪服务，严查投诉问题，不断提高项目落地率和资金到位率。

　　加快重点项目建设步伐。在全力抓好16项在建市级领导干部包抓重点项目的同时，确保今年27项重点项目如期开工。尤其要围绕关城大景区5大项目、立体交通综合体系、多功能专用汽车产业园区、市第一人民医院、中医院综合楼、棚户区改造工程等重大项目建设，凝心聚力、攻坚克难，抓工期、抢进度，确保尽早建成见效。积极做好28项重点项目前期工作，争取2018—2020年陆续开工建设。

　　激发民间投资活力。积极争取国家政策和资金支持，推进政府与社会资本合作（PPP）、政府购买服务等新型投融资模式，鼓励民营资本参与到城市公用设施、道路交通、市场改造等特许经营项目建设运营，支持民间资本进入医疗、养老、教育等民生领域，促进民间投资回稳向好。全面落实省委新发展理念“1+19”配套政策、市委促进非公有制经济加快发展试行办法“25条”，切实降低民间资本准入门槛，促进各类市场主体公平竞争，营造重商、亲商、安商的发展氛围，充分激发非公有制经济发展活力。

　　（七）聚焦精细管理，提升城市文明形象。按照产城一体、景城一体、城乡一体发展理念，统筹协调城市产业和空间布局，不断提升城市综合服务功能和文明形象。

　　强化城市规划引领，加强“多规合一”，推进城市总体规划和土地利用总体规划“两图合一”，实现一张蓝图干到底。着力补齐城市文明短板，有序推进棚户区改造与老旧住宅小区综合整治，累计投资8.83亿元，对绿化、祁峰建化、嘉峪关化工总厂、原百货站家属院、胜利北路单身宿舍5个小区2342套住房进行改造，力争8月开工建设，2018年9月交付使用；进一步提高棚户区改造货币化安置率，并与人口城镇化和城乡一体化建设有机结合，促进房地产库存持续下降。统筹规划城乡各类市场改造提升和新建工作，全面加快集贸市场改造提升步伐。结合创建全域旅游城市，统筹规划城乡公厕建设，切实加大管理水平。科学论证、适时推进全市城市地下综合管廊建设，大力推广装配式建筑。

　　推进城市治理体制机制创新。着力实施“雪亮工程”，整合完善公共安全领域视频资源，实行统一管理应用。加强网格化服务管理，将生态保护、环境卫生、综合治理、安全生产等工作纳入网格，完善网格员工作职责，强化“12345”民生热线职能，切实加大巡察、转办、督查力度，加快形成常态化的城市治理模式。着力推进智能交通系统建设，科学设置道路交通安全基础设施，规范城市停车设施规划建设与管理，构建文明畅通安全的道路交通秩序。广泛深入开展“全域全城无垃圾”创建活动，努力形成“城乡社区主抓、群众积极参与、单位全力配合”的共治共享格局。加快推动城市管理地方立法，逐步实现城市管理法治化。推进城市管理体制改革、水务一体化改革、城乡生态绿化管理一体化改革，吸引社会力量和社会资本参与城市管理服务，深化环卫保洁、园林管护等领域市场化改革，推动公用基础设施建设管理市场化、专业化、社会化。

　　力争成功创建全国文明城市。坚持把创建工作作为一项利民之策、惠民之举，严格对照测评的40项一级指标、90项二级指标，聚焦市容市貌、交通秩序、基础设施、市场治理、窗口服务、未成年人教育等薄弱环节，认真落实2017年创建全国文明城市工作实施方案，更加注重抓细节、补短板、强素质，举全市之力打赢创建攻坚战。

　　（八）坚持生态立市，促进绿色低碳发展。贯彻落实绿色发展理念，加大环境保护力度，巩固提升园林绿化建设成果，加快节能型城市建设步伐。

　　坚持增绿与提质相结合，拓展绿色空间，提高绿地管养水平和质量。抓好市区道路绿化提升改造，完善绿化配套设施，形成一路一景、风格多样的绿化格局，重点实施迎宾湖旅游园区基础设施、城市立体绿化、图案绿化等工程和文化路、胜利路、建设路等10条道路绿化带的提升改造；大力开展花园式单位创建和农村庭院、村庄“四旁”绿化活动，加强对新建居住区的绿化监管，巩固提高绿化成果。

　　树立抓环保就是抓发展、抓环保就是促转型的理念，努力让天更蓝、水更绿、城更美，让广大市民和游客在城市生活得更方便、更舒心、更美好。全面落实大气、水、土壤污染防治“三大行动”计划和最严格水资源管理制度考核办法，实施“蓝天、碧水、净土”工程，持续推进工业企业达标治理、燃煤锅炉综合整治、扬尘污染防治、黄标车及老旧车辆淘汰、餐饮油烟和煤炭污染治理；保障饮用水源地水质安全，强化工业企业污水治理，加大城镇水污染、畜禽养殖污染防治力度；推进工业企业污染场地等重点区域土壤污染治理与修复试点工作，加强核与辐射安全监管，切实保障辐射环境安全。严格执行国家《生态文明建设目标评价考核办法》，狠抓环境执法监管，始终保持严厉打击环境违法行为的高压态势，全面落实环境保护“党政同责、一岗双责”责任制。做好全国第二次污染源普查前期准备工作。进一步加大节能减排、清洁生产力度，巩固工业园区循环化试点建设成果。大力发展绿色建材，加快先进技术的应用，全力推进公共机构节能型示范单位创建工作。

　　（九）持续改善民生，提高人民幸福指数。坚持以人为本、民生优先，编密织牢民生保障安全网，不断提升人民群众的获得感和幸福感。

　　稳步提高社会保障水平。加强就业指导和服务，多措并举兜住就业底线，推进和谐劳动关系建设，严肃查处农民工欠薪违法行为。完成全民参保登记，摸清社会保险家底，扩大社会保障覆盖面，建成“五险合一”信息系统，实现城乡养老“一卡通”；不断拓展医疗异地就医直接结算范围和对象，进一步提高城乡低保、五保供养、残疾人和医疗救助水平。

　　优先发展教育事业。实施“名师名家工程”、教师素养提升工程，全面加强师资队伍建设；全面落实大力促进学前教育改革与发展实施办法“31条”，加大普惠性民办幼儿园奖补力度，不断提高幼师专业水平，全面普及学前教育；加快职业教育资源整合进程，推进甘肃省冶金高级技术学院、职教中心与上海交大教育集团合作步伐，积极建设中德学院，提升职业教育服务地方发展能力；加快实施胜利路小学、实验中学、师范附校初中部改扩建项目，建成福民、建林棚改区幼儿园，推进南市区小学及幼儿园、市酒钢三中体育馆、甘肃钢铁职业技术学院实训基地建设。实施健康行动计划，协调推进医疗、医保、医药“三医”联动改革，完成城乡居民医保制度整合，建立城乡统一的居民医保制度；落实分级诊疗制度，建立健全医疗资源下沉长效机制，推动城市综合医疗机构与社区、镇村医疗卫生机构之间形成“大手拉小手”的紧密型医联体建设；实施“名医生名护士”和医护人员素养提升工程，鼓励建立名医工作室，切实加强医疗卫生队伍建设。

　　推进文化惠民工程，扩大乡村舞台、文化大院、文体广场覆盖范围，继续打造雄关大舞台等文化品牌。统筹建设全民健身公共服务体系和产业链、生态圈，推进全市各类公共体育设施向广大市民开放。

　　继续办好惠民实事。坚持普惠性、均等化、可持续方向，围绕解决人民最关心、最直接、最现实的急难愿盼问题，筹措资金10.89亿元，在老城区道路改造及绿化提升、老旧小区基础设施综合改造、城乡公交线路优化与延伸服务、城乡停车设施规划建设与管理、城乡市场规划建设与改造、持续补齐医疗卫生短板、扶持学前教育发展、亮化城乡结合部及三镇农村主干道、推进全民体育健身工程、发放部分特困家庭基本生活补贴方面，全力为民办好10件实事。

　　（十）强化风险防控，促进社会和谐发展。主动应对和科学管控各类风险，促进经济平稳健康发展和社会大局稳定。

　　防范和化解金融领域风险。落实“属地管理”金融风险防范和财政风险责任，建立定期风险排查研判和协同处置机制，严厉打击非法集资；严格执行重大项目投资、大额资金使用决策制度，遵循“量入为出”原则，正确处理加大项目建设力度与财政承受能力的关系，注重项目可行性论证，加强政府融资平台管理，建立健全风险评估、预警和应急处置机制。

　　维护社会和谐稳定。坚持人民利益至上，进一步畅通信访渠道，有效化解社会矛盾。严格落实“党政同责、一岗双责、失职追责”责任体系，切实加强对生产安全、环境安全、食品药品安全、特种设备安全等重点领域的隐患排查和专项整治，坚决守住安全红线，不断增强安全防范治理能力。推进国家食品安全示范城市和农产品质量安全市创建工作。全面构建立体化社会治安防控体系，建好用好市、区、社区三级综治中心，优化整合三大中心职能，推动公安实战化指挥中心、情报中心和智能交通指挥中心建设，加快智慧社区、标准化派出所建设进程，提升社会治理能力。加强诚信体系建设，通过法治、行政手段联合惩治失信行为，维护社会正常秩序和群众合法权益。

　　三、打造务实高效政府

　　完成今年目标任务，对政府自身建设提出了新的更高要求。我们要认真贯彻党的十八届六中全会精神，进一步巩固拓展“两学一做”学习教育成果，把全面从严治党要求贯彻落实到政府各项工作中，切实增强“四个意识”，全面提升执行力、公信力。

　　一是提升决策水平。要加强对新理念、新知识和新方法的学习，努力提升研究政策、运用政策和落实政策的能力，把我们想干的事情纳入国家和全省的支持范围。要紧紧围绕市委“实践第一、大抓基层、群众至上”三个导向，将调查研究作为基本的工作方法、工作方式和工作能力，不断深化对市情的认识和把握，切实做到工作情况明、底数清、数字准，确保决策依据真实、措施办法精准，力戒形式主义、做表面文章和弄虚作假。要把法治思维和法治方式贯穿于政府工作全过程，严格全面贯彻落实新修订的市政府工作规则，成立市政府专家咨询委员会，推进法治政府建设，坚持政府法律顾问制，加强政府立法，把公众参与、专家论证、风险评估、合法性审查和集体讨论决定作为重大行政决策的法定程序，不断提高科学、民主、依法决策能力和水平。

　　二是强化落实能力。牢固树立“一盘棋”意识，努力做到上下左右协调联动，始终保持政令畅通，切实形成同频共振、合力攻坚的工作局面。要靠实责任抓落实，聚焦经济社会发展突出问题，对照重点工作部署和量化指标任务，逐项分解细化，逐级明确责任，层层传导压力，确保各项工作按时间节点有序推进。要强化举措抓落实，以“互联网+政务服务”为牵引，着力推行网上政务大厅、手机政务APP等网上理政措施，推动“放管服”改革措施落地；加大督查力度，创新督查方式，严格考核问效，坚持激励问责并举，建立健全正向激励机制和容错机制，有效激发广大干部干事创业热情。要改进作风抓落实，把对上负责与对下负责有机地结合起来，以开展“企业服务年”活动为契机，实实在在沉下身子，认认真真倾听群众心声，大力发扬脚踏实地、埋头苦干的作风，持续优化发展环境，“撸起袖子加油干”。

　　三是加强廉政建设。要严格落实从严治党主体责任，坚持把纪律和规矩挺在前面，用铁的纪律加强和规范政府各项工作。要紧扣“六个关键环节”完善机制，强化“六项监督”，牢固树立“过紧日子”思想，严控“三公”经费，严管公共资金资产资源，全面推进行政决策、执行、管理、服务、结果“五公开”，让权力在阳光下运行。要切实履行好党风廉政建设“3783”主体责任，着力整治庸政懒政怠政行为和不担当、不作为、慢作为等突出问题，严肃查处各类违纪违法案件，努力建设为民、务实、清廉、高效政府。

　　各位代表，做好今年政府工作，任务艰巨、责任重大。让我们紧密团结在以习近平同志为核心的党中央周围，在省委、省政府和市委的坚强领导下，团结拼搏，真抓实干，攻坚克难，奋发有为，为实现“两个率先”目标而努力奋斗，以优异的成绩迎接党的十九大和省第十三次党代会胜利召开！
